# Supplementary material for: The interactome of intact mitochondria by cross-linking mass spectrometry provides evidence for coexisting respiratory supercomplexes
Source: Mol Cell Proteomics. 2017 Dec 8;17(2):216–32. doi: 10.1074/mcp.RA117.000470 (PMC5795388; doi:10.1074/mcp.RA117.000470)
Supplement: Supplemental Data [file supp_RA117.000470_133922_0_supp_23978_fzffwf.zip › spectra_annotation/mito_DR_spectra_annotation/153-1-6-1-24-1.pdf]

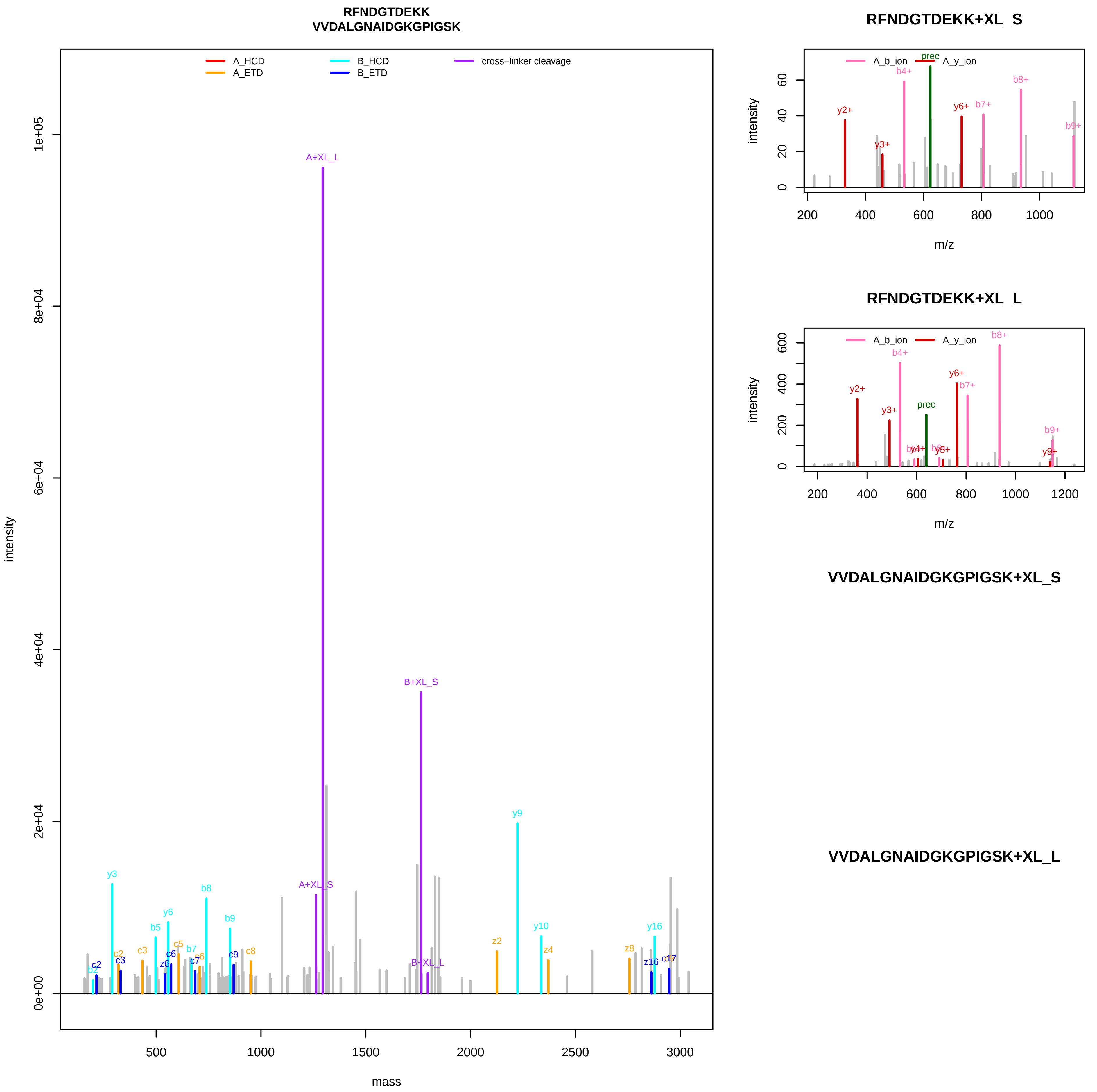
RFNDGTDEKK
VVDALGNAIDGKGPIGSK
A_HCD
A_ETD
B_HCD
B_ETD
cross−linker cleavage
A+XL_L
B+XL_S
A+XL_S
B+XL_L
y3
b5
y6
b8
b9
y9
y10
y16
c2
c3
c5
c6
c7
c8
c9
c17
z2
z4
z6
z8
z16
intensity
mass
RFNDGTDEKK+XL_S
A_b_ion
A_y_ion
prec
b4+
b7+
b8+
b9+
y2+
y3+
y6+
m/z
RFNDGTDEKK+XL_L
y4+
y5+
y9+
VVDALGNAIDGKGPIGSK+XL_S
VVDALGNAIDGKGPIGSK+XL_L
